# Artificial Intelligence and Objective Structured Clinical Examinations: Using ChatGPT to Revolutionize Clinical Skills Assessment in Medical Education

**DOI:** 10.1177/23821205241263475

**Published:** 2024-07-25

**Authors:** Sanghamitra M. Misra, Srinivasan Suresh

**Affiliations:** 1 Division of Academic General Pediatrics, Department of Pediatrics, 3989 Baylor College of Medicine, Houston, Texas, USA; 2 3984 Texas Children's Hospital, Houston, Texas, USA; 3 Divisions of Health Informatics & Emergency Medicine, Department of Pediatrics, 12317 University of Pittsburgh School of Medicine, Pittsburgh, Pennsylvania, USA; 4 6619 UPMC Children's Hospital of Pittsburgh, Pittsburgh, Pennsylvania, USA

**Keywords:** Artificial Intelligence, clinical competence, test anxiety, medical education, checklist

## Abstract

This article examines the integration of OpenAI's Chat Generative Pre-trained Transformer (ChatGPT) into Objective Structured Clinical Examinations (OSCEs) for medical education. OSCEs, essential in evaluating medical trainees, are time and resource-intensive for educators and medical colleges. ChatGPT emerges as a solution, aiding educators in efficient OSCE preparation, including case development, standardized patient training, assessment methods, and grading rubrics. We explore ChatGPT's role in reducing trainee stress through simulated interactions of realistic practice scenarios and real-time trainee feedback. We also discuss the importance of validating ChatGPT outputs for medical accuracy and address compliance concerns. While highlighting ChatGPT's potential in reducing time and cost burdens for educators, we underscore the need for careful and informed application of Artificial Intelligence in medical education. Through examples, we outline ChatGPT's promising future in augmenting medical training and assessment, balancing technological innovation with educational integrity.

## Introduction to Objective Structured Clinical Examination

Objective Structured Clinical Examinations (OSCEs) are timed, standardized skills assessments used world-wide and across specialties by medical colleges to help medical students and residents improve competence and confidence in clinical skills.^1^ OSCEs are fundamental in helping trainees become successful clinicians. Originally created in the mid-1970's, they are fundamental in providing real-time formative feedback and summative grades, and they may be administered in face-to-face, virtual, and hybrid platforms.^2^ OSCEs mimic real-world encounters in a controlled environment, so educators can identify gaps in a trainee's performance and provide specific, effective feedback.^3^ OSCEs can be dynamic/observed where a standardized patient (SP) actively interacts with the trainee posing as a patient or caregiver of a patient, or they can be static/pseudo-OSCEs where knowledge is assessed at stations with written or online questions.^4^ With increased telemedicine use over the last decade and specifically with the COVID-19 pandemic, educators have increased focus on teaching and assessing telephonic and virtual medicine curricula through OSCEs.^5,6^ OSCEs are also used by educators in other health professions.^7–9^

OSCEs require substantial preparation by educators to create high-quality, successful, and validated assessments of students and residents.^4^ It is difficult to achieve standardization within OSCEs given natural variation in SP and student moods, previous student knowledge or experience with the specific diagnoses, complexity of cases, and level of SP preparation. However, the use of multiple cases can improve fairness and validity of an OSCE exam.^1^ Therefore, educators often search for previously validated OSCEs to improve formative and summative assessment of their students. In fact, in the peer-reviewed medical education repository MedEdPORTAL, 188 entries include the word OSCE.^10^ Identifying ways to help educators more efficiently create and administer OSCEs could help improve overall educator satisfaction.

Although trainees are prepared throughout a course or program to perform well on an OSCE, many trainees feel stress and anxiety because of the high-stakes nature of OSCE exams. Due to the wide range of clinical presentations that may be assessed during the exam, students frequently express concerns about exam readiness. Platforms that allow live OSCE practice could improve OSCE readiness and reduce test anxiety.

## Introduction to Chat Generative Pre-trained Transformer

OpenAI's Chat Generative Pre-trained Transformer (ChatGPT)^11^ is a web-based conversational agent, an Artificial Intelligence (AI)-driven large language model (LLM). It operates via its online platform,^12^ delivering responses that closely mimic human conversation. Responses, predominantly in text format, are not restricted to the healthcare sector. The capabilities of GPT-4, which is ChatGPT's latest model, include a range of tasks like instant language translation and analyzing emotional tone. ChatGPT is constructed on an expansive framework of 175 billion parameters, a number that continues to increase,^13^ enabling it to cover a broad spectrum of topics and engage in detailed dialogs according to user inputs. Fundamental to LLMs like ChatGPT is the ability to produce conversational text. LLMs achieve this by analyzing patterns in a massive collection of textual data, using deep learning architectures that mimic the complex structure of the human brain. Since December 2022, ChatGPT has been used by educators and trainees across the world. Interestingly, ChatGPT's performance on a real OSCE was tested in early 2023. On an obstetrics and gynecology OSCE, ChatGPT received a higher score from 14 human examiners (average 77.2%) compared to scores from 26 learners (average 73.7%). Notably, ChatGPT finished each 10-min station in approximately 2.54 min, and many examiners were unable to determine which responses were from learners and which were from ChatGPT.^14^ In medical education, the use of AI, specifically ChatGPT, requires caution due to potential inaccuracies in AI-generated content. ChatGPT's performance on standardized undergraduate medical education and medical specialty exams has been documented, and although impressive, it is not yet perfect.^15–17^ Trainees must not rely solely on ChatGPT's medical accuracy for acquiring knowledge and exam preparation. However, when guided by trained, expert medical educators, AI can be a valuable tool to augment medical education. The application of AI in OSCE preparation, though promising,^18^ remains in early development stages.

## ChatGPT applications for OSCE preparation by medical educators

OSCEs require substantial educator preparation time to identify gaps in student assessment within a course/program, create appropriate case scenarios to fill those gaps, design all necessary details of cases, create static stations, develop SP guides, create assessment tools to assess student performance accurately, and provide meaningful feedback to trainees. As medical education rapidly incorporates evolving technologies, it is to the benefit of medical educators to improve OSCE creation efficiency. AI can provide an impressive boost to an educator's ability to create OSCE content and assessments rapidly.^19^ It should be also noted that OSCEs usually require funding to administer, and the use of ChatGPT may help reduce the cost burden to the college by reducing personnel time needed to administer the exams. Table 1 summarizes ChatGPT's potential contributions in creation and administration of OSCEs and notes current limitations. Educators should gain familiarity with this new technology, especially as it gains widespread acceptance.

**Table 1.** Capabilities of ChatGPT in aiding educators and trainees with OSCE preparation.

EDUCATOR CURRICULAR NEED	ChatGPT CONTRIBUTION	ChatGPT LIMITATIONS
Determine assessment gaps of clinical skills within a course or program	Review a syllabus to determine areas that need more robust clinical skills assessment	Limited published standardized recommendations for courses to be used for comparison
Determine assessment needs for individual students or cohorts of students based on evaluation data	Analyze student performance on exams and review comments from written evaluations to determine areas of weakness that may benefit from a specifically designed OSCE	Input for analysis must be de-identified as ChatGPT is not FERPA compliant^20^
Full cases based on course objectives	Create full case scenario based on patient age and diagnosis with common lab results^3^ and post-encounter notes	Needs clinician review for medical accuracy
Questions for static stations	Create cases and questions based on course and level of trainee	Limited ability to create graphics/medical images
SP guide	Case explanation with full details about patient Q&A examples Can pose as student and help with SP training	Needs clinician review for medical accuracy
SP checklists for assessment	Create checklist based on number of questions requested and points of importance	Needs educator review for appropriateness and accuracy
Communication assessment	Checklist for SPs or reviewers to use assessing patient understanding	Needs educator review for appropriateness and accuracy
Grading rubric	Create points system to grade a summative OSCE including history-taking accuracy, clinical reasoning, communication skills, and other relevant categories	Unable to provide support for hands-on physical exam assessment-clinician must observe and provide feedback^21^
Student performance feedback	Provide detailed assessments, explanations, and summaries of student OSCE transcripts	ChatGPT is not FERPA compliant,^22^ so input must be de-identified.

ChatGPT, Chat Generative Pre-trained Transformer; SP, standardized patient; FERPA, Family Educational Rights and Privacy Act.

### Identifying gaps in clinical skills assessment within a program

The first step of OSCE development involves pinpointing gaps in student clinical skills assessment, aligned with program objectives and existing assessment methods. While ChatGPT can analyze a syllabus to identify unassessed clinical skills, the lack of standardized medical education courses and the challenge of uploading all measures into ChatGPT limit its effectiveness. Additionally, uploading student performance data to detect knowledge gaps in clinical skills faces hurdles due to Family Educational Rights and Privacy Act (FERPA) regulations and data formatting issues. These constraints suggest ChatGPT's limited utility in the early stages of OSCE preparation.

### OSCE case development

Upon identifying a clinical skill assessment need, a medical educator designs an OSCE case encompassing presentation, history, physical exam findings, labs/imaging, clinical reasoning, assessment, and plan. ChatGPT significantly aids this process. It rapidly generates customized cases when prompted with specifics like diagnosis and patient age. Through interactive dialogue, cases are refined to suit educational goals. ChatGPT's capacity to instantly create diverse cases from simple prompts is vast, yet educators must ensure clinical accuracy and trainee-level appropriateness. Beyond full case creation, ChatGPT assists in formulating static station questions aligned with course objectives. For example, it can generate questions to evaluate a resident's knowledge of acute heart failure treatment or a medical student's grasp of status asthmaticus management.

### Creation of SP trainee assessment checklist

An important part of an SP's time and expertise is assessing a trainee's performance after a clinical interaction. In general, an SP marks a checklist to document whether a trainee asked specific questions and if communication skills were appropriate and effective. For an existing case or a new OSCE case, ChatGPT can create an SP checklist based on the specific patient presentation, case details, areas of focus during history-taking, number of items requested, and any unique focus points based on the distinct case presentation. Again, ChatGPT can continue to modify the checklist based on feedback from the educator until it is satisfactory.

### SP training

SP training is a time-intensive task. The training is generally the shared responsibility of the medical educator and SP administration team. SPs are given a script with case details that must be put to memory. The SP role is challenging, because students ask a variety of questions in different ways, and sometimes, it is difficult for SPs to remain calm, answer the questions accurately based on the memorized script, and assess the student's performance simultaneously. ChatGPT can create mock-OSCEs, posing as a student and engaging in a clinical interaction with the purpose of training the SP. Without an educator or SP administrative team member present, an SP can engage in practice sessions to prepare themselves for the actual exam. ChatGPT is capable of grading SP checklists from OSCE transcripts, freeing up SP time to provide more in-person real-time detailed trainee feedback or to engage in more student OSCE encounters within a given exam period.^23,24^ And if allowable by the college or program, ChatGPT may even remove the need of identifying and training SPs by assuming the SP role in an OSCE encounter.

### Assessment, grading, and feedback

In OSCEs, the assessment method, grading rubric, and quality of feedback are crucial for a successful exam. ChatGPT can develop tailored assessment methods like post-encounter notes and case-specific grading rubrics. By analyzing sample assessments used by educators or institutions, ChatGPT can generate similar evaluations. Additionally, LLMs can provide in-depth analysis of student OSCE transcripts. Students often seek extensive feedback during clinical interactions and OSCEs. Detailed OSCE score reports are essential for students to identify their strengths and weaknesses, helping them develop strategies to enhance their overall competence and performance.^25^

## ChatGPT contribution example

**Figure 1.**
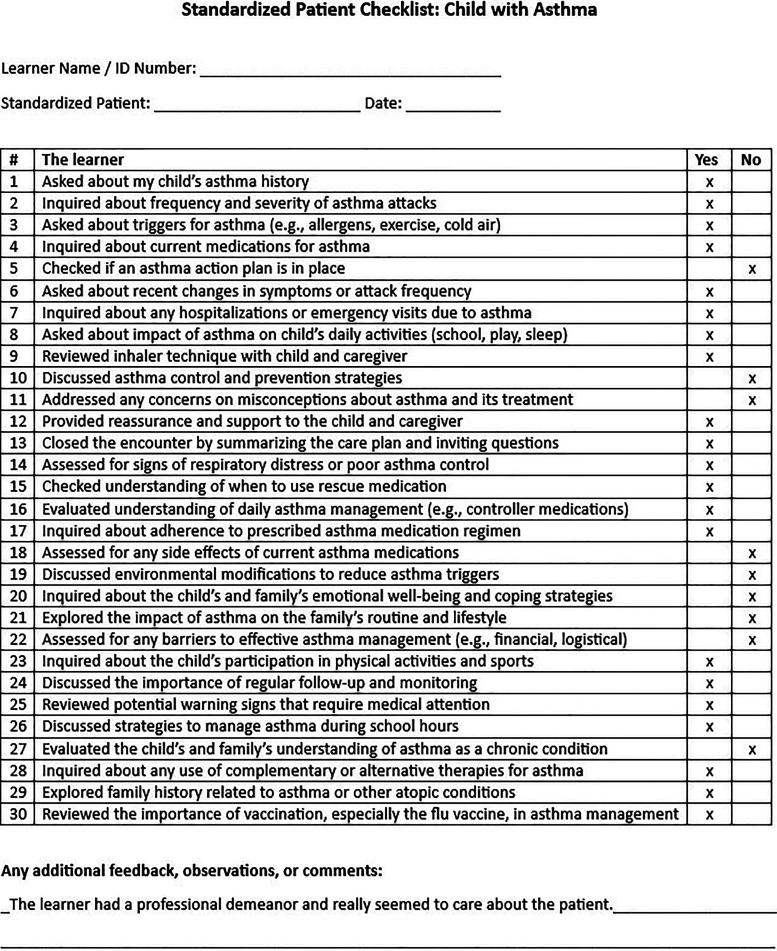
ChatGPT generated standardized patient checklist for an OSCE session for asthma with randomly marked sample assessment. ChatGPT, Chat Generative Pre-trained Transformer.

**Figure 2.**
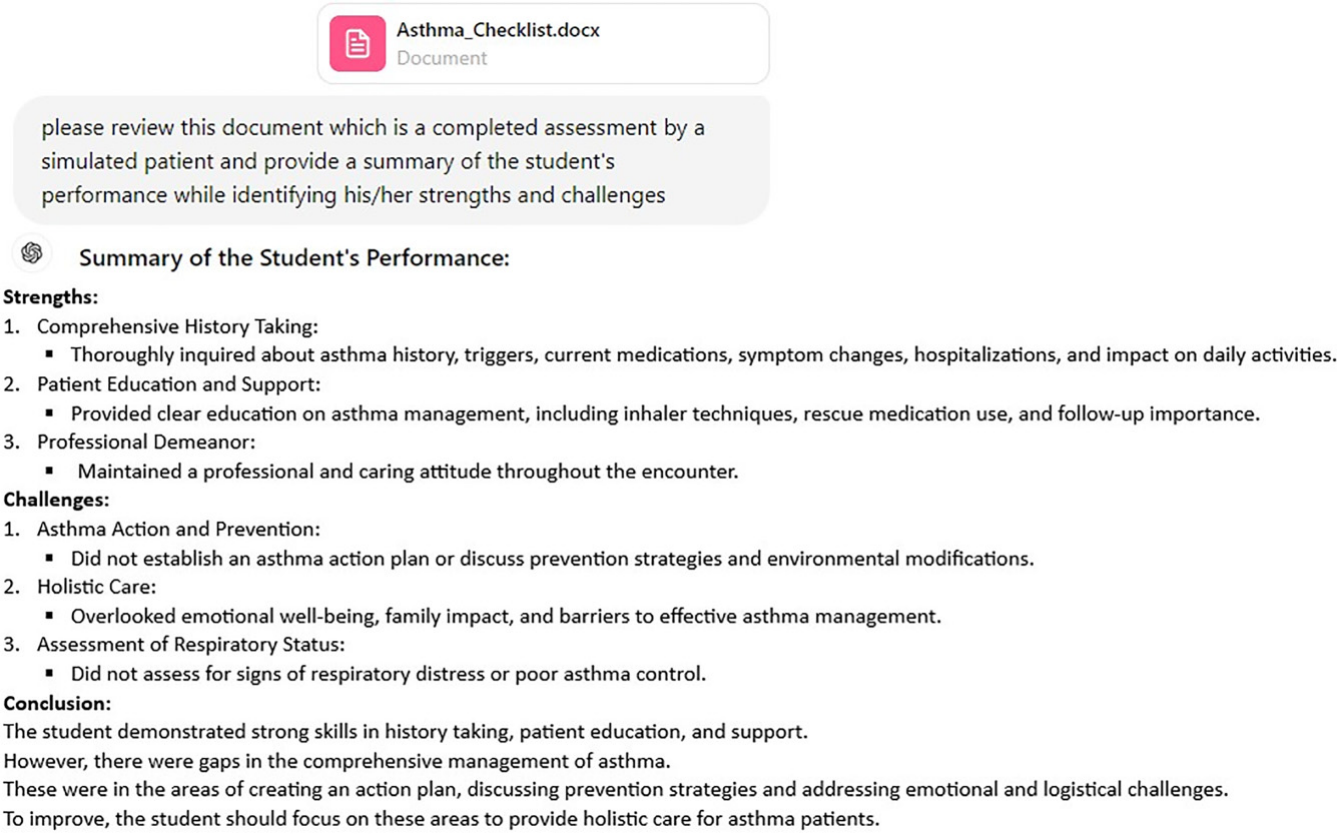
ChatGPT summary of trainee performance. ChatGPT, Chat Generative Pre-trained Transformer.

To demonstrate the capabilities of ChatGPT in generating an OSCE standardized checklist, we first uploaded a validated SP checklist from a published OSCE on neonatal jaundice.^26^ We then prompted ChatGPT, based on the previously uploaded jaundice checklist, to create a new OSCE checklist for a child with asthma, and it generated a 30-question checklist. By randomly assigning “yes” or “no” to each line on that checklist, we created a sample completed assessment (Figure 1) and uploaded the document into ChatGPT, following which we prompted for a trainee's performance summary (Figure 2). The ChatGPT summary provided a comprehensive assessment of the trainee's performance highlighting strengths and challenges.

## Use of ChatGPT in non-traditional OSCEs

ChatGPT has been used to improve emergency medicine (EM) physicians’ ability to break bad news.^27^ Although traditionally reliant on SP scenarios, this new approach utilizes ChatGPT to create realistic clinical scenarios for active role-play and feedback. Using specifically ChatGPT-3.5, a detailed prompt created a realistic training scenario, with ChatGPT acting as the patient and providing feedback based on the SPIKES framework (Setting up, Perception, Invitation, Knowledge, Emotions with Empathy, and Strategy or Summary). This novel method shows potential for AI in medical education in general and specifically in the training of EM physicians.

ChatGPT can help create, administer, and assess OSCEs across medical specialties which assess a wide variety of traditional clinical skills as well as newer skills that have been incorporated into medical curricula. OSCEs can assess communication skills on social determinants of health concepts such as environmental exposures, food insecurity, access to care, and access to housing. OSCEs can also help students improve their communication with patients with limited English proficiency with an exam that includes an SP and an interpreter. ChatGPT can create interpreter OSCEs based on medical specialty.^28^ AI-driven OSCEs can be particularly beneficial for key areas of training that are difficult to assess with written exams such as obtaining informed consent for elective surgery or blood product administration.

## Trainee OSCE preparation

ChatGPT has demonstrated the ability to help students and residents prepare for OSCE exams by building specific trainee-level case scenarios and providing real-time feedback to improve communication and clinical reasoning skills.^29^ ChatGPT can create cases based on a trainee's course (pediatrics, ob-gyn, etc), and it can provide specific feedback on important communication skills such as limiting medical jargon. It should be noted that ChatGPT may be too encouraging and not provide critical feedback, so trainees must guide ChatGPT to optimize feedback received.^30^ Also, as mentioned above, ChatGPT does not always provide medically accurate answers to medical questions, so trainees should not use ChatGPT output specifically to increase medical knowledge. Interestingly, AI-based platforms like Geeky Medics and OSCELab are already preparing students for OSCEs with their interactive online cases.^31,32^ Student anxiety and stress during OSCE examinations is a known phenomenon, and peer-led practice OSCEs have been beneficial in improving trainee confidence specifically for summative OSCEs.^20,33^ Practicing OSCEs with AI guidance may help reduce test anxiety because students feel better prepared.^20,25,29,34^ As we allow trainees to use ChatGPT to help with tasks such as OSCE preparation, it is imperative that educators emphasize the importance of medical ethics related to AI use with existing frameworks such as the principle-based approach of public health ethics.^35^

## Conclusion

OSCEs are valuable assessments in medical education for trainees at all levels, but they require significant amounts of time for creation and administration. ChatGPT's integration into medical education, particularly in OSCEs, marks a significant advancement. Its ability to aid educators in creating and administering these examinations efficiently while providing trainees with an innovative platform for practice and feedback is noteworthy. Additionally, ChatGPT can help with valuable SP training. However, it is crucial to approach this technology with caution, ensuring medical accuracy and adherence to regulations like FERPA. As ChatGPT continues to evolve, its potential to revolutionize medical education is undeniable, promising a future where AI complements traditional teaching methods in addressing curricular needs such as communication assessments and OSCE grading rubrics. ChatGPT will undoubtedly influence the training and assessment of future medical professionals. Medical educators should strive to understand appropriate AI use in their own teaching, and they should serve as role models and guides for trainees using AI as a part of their education.
